# Analysis of cell identity, morphology, apoptosis and mitotic activity in a primary neural cell culture system in *Drosophila*

**DOI:** 10.1186/1749-8104-7-14

**Published:** 2012-06-20

**Authors:** Manuela M Moraru, Boris Egger, Diarra B Bao, Simon G Sprecher

**Affiliations:** 1 Institute of Cell and Developmental Biology, Department of Biology, University of Fribourg, Chemin du Musée 10, Fribourg, 1700, Switzerland

**Keywords:** *Drosophila*, Neurogenesis, Notch, Primary brain culture, Neural cells

## Abstract

In *Drosophila,* most neurogenetic research is carried out in vivo. Mammalian research demonstrates that primary cell culture techniques provide a powerful model to address cell autonomous and non-autonomous processes outside their endogenous environment. We developed a cell culture system in *Drosophila* using wildtype and genetically manipulated primary neural tissue for long-term observations. We assessed the molecular identity of distinct neural cell types by immunolabeling and genetically expressed fluorescent cell markers. We monitored mitotic activity of cell cultures derived from wildtype and tumorous larval brains. Our system provides a powerful approach to unveil developmental processes in the nervous system and to complement studies in vivo.

## Background

Neurogenesis in *Drosophila* is a biphasic process consisting of an embryonic and a postembryonic period of neurogenesis. During embryogenesis primary neurons are generated, that form the functional larval central nervous system (CNS). Subsequently, during postembryonic neurogenesis secondary neurons are generated, which build up the adult brain during larval and pupal stages. During embryonic stages neural precursor cells termed neuroblasts (NBs) divide asymmetrically in a stem cell-like fashion thereby self-renewing and producing smaller ganglion mother cells (GMCs). The GMCs have a limited mitotic potential and divide only once more, to generate a pair of neurons and/or glial cells. At the end of embryogenesis, NBs undergo a quiescent phase and only a subset of NBs enter mitosis again to generate secondary neurons during larval development, reviewed in [1-3].

Depending on their mode of proliferation, larval NBs can be further subdivided into Type I and Type II NBs. In contrast to Type I NBs, in which the GMCs divide once to form two postmitotic cells, Type II NBs give rise to an intermediate progenitor that can divide multiple times. Therefore, Type II lineages are substantially larger than Type I lineages [4-6]. A third type of neurogenesis occurs in the developing optic lobe, where NBs derive from neuroepithelial precursors. Neuroepithelial cells initially divide symmetrically to increase the pool of precursor cells. Later, during larval development, neuroepithelial cells gradually transform to NBs and switch to an asymmetric division mode [7].

In contrast from what we know about mammalian neural stem cell behavior, most knowledge about precursor cells in the *Drosophila* nervous system is based on findings in vivo. It has been difficult to study defined neural *Drosophila* cells in vitro over a longer culture period [8]. Dissociation of neural tissue into individual cells allows studying how neural precursors, differentiating neurons and glial cells behave outside their natural environment and therefore, to determine what aspects are controlled by either extrinsic or intrinsic cues. Many processes in the development of the brain depend on signals from neighboring cells and the correct environmental context. For instance, the reactivation of NBs in early larval stages is regulated through paracrine signals, *Drosophila* insulin like peptides that derive from glia cells [9,10]. In contrast, an intrinsic cascade of transcription factors regulates the temporal identity of NBs and the fate of their progenies. This process occurs independently of the cellular environment, since the temporal cascade is not altered in isolated NBs in culture [11].

Development and neurogenesis in the CNS underlie tightly controlled molecular mechanisms, to ensure that the correct number and types of neurons are generated at different developmental stages. There are two main mechanisms to terminate NBs proliferation in the larval CNS. In the central abdomen pro-apoptotic proteins are activated at advanced larval stages to remove the dividing NBs by cell death once the neuronal lineage is complete [12]. Thoracic and central brain NBs, instead, proliferate two days longer into pupal stages and then make a final symmetric division before exiting the cell cycle [13]. An intriguing question is whether the termination of NBs proliferation is solely controlled by intrinsic factors or whether extrinsic mechanisms have any role in this process.

We investigate the behavior of neural cells outside their endogenous environment in a newly developed primary cell culture system for larval CNS cells. We use cell type specific molecular markers in order to identify neural precursors, differentiating neurons and glial cells in vitro. Genetically controlled expression of fluorescent markers allows us to identify specific subtypes of cultured brain cells. We further investigate the mitotic activity of brain cells after dissociation. We show distinct labeling techniques to identify mitotically active cells and cells that undergo apoptosis in vitro. Finally, we demonstrate that our primary culture system can maintain proliferating optic lobe precursor cells.

## Results and discussion

### Identifying neuronal and glial cells in primary cell culture

**Figure 1:**
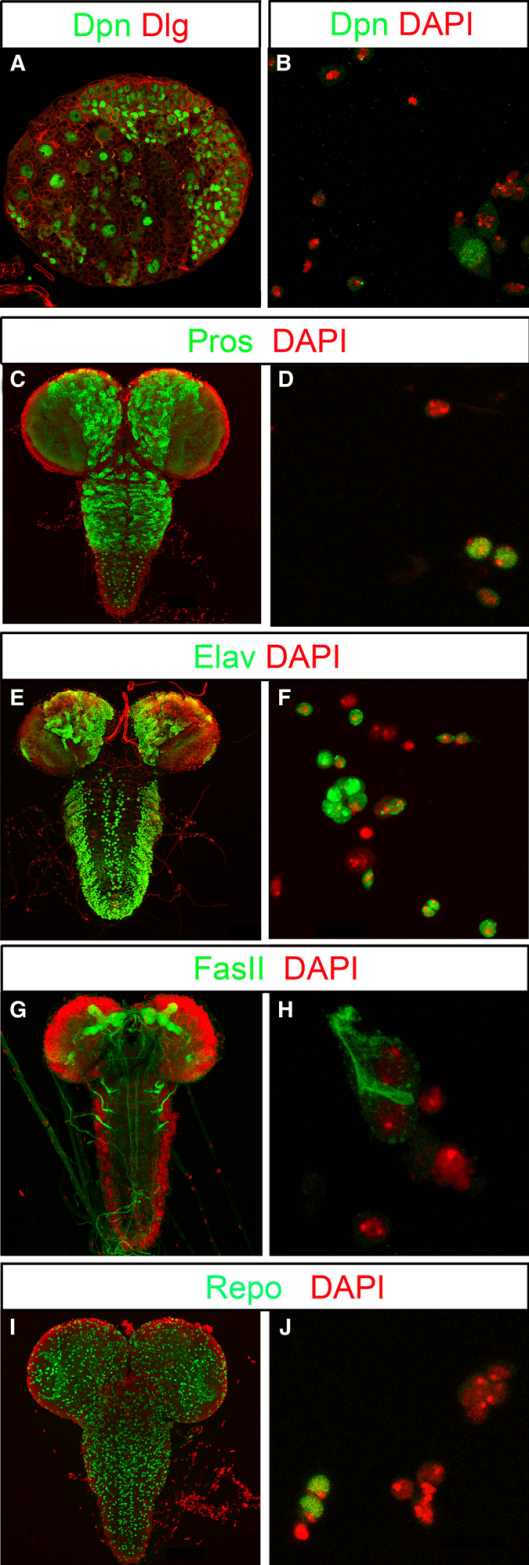
**Molecular markers of neural cells in third instar larval brains and primary cell cultures.** Immunostainings are displayed together with the DNA-marker DAPI (red) to label the nuclei of all cells. **(A)** Shows one brain hemisphere, anti-Dpn (green) labels nuclei of NBs, Dlg labels cell cortices (red in A). **(B)** Shows Dpn-labeled progenitors in primary brain cultures. **(C, D)** Anti-Pros (green) stains the nuclei of both GMCs and neurons. **(E, F)** Anti-Elav (green) marks nuclei of postmitotic neurons. **(G, H)** In the brain anti-FasII labels pioneering axonal fascicles, in cultured neurons anti-FasII marks membranes of pioneering neurons. **(I, J)** Glial cells within brains and culture are marked by anti-Repo (green). All images are Z-projections of confocal stacks.

Neural precursor cells and differentiated neuronal cell types in the *Drosophila* larval brain can be identified by the expression of unique molecular markers through immunohistochemistry. In order to test whether these same markers can be used in primary cell culture, we dissociated wandering third instar larval brains. All NBs in the central brain, ventral nerve cord (not shown) and the optic lobes express the Hes type transcription factor Deadpan (Dpn) [14] (Figure 1A). We can detect Dpn expression in primary cell culture in large precursor cells but not in more differentiated cell types (Figure 1B). The homeodomain transcription factor Prospero (Pros) marks the nuclei of GMCs and undifferentiated neurons in the larval brain [15-17] (Figure 1C). In the primary culture, Pros can be detected in nuclei of smaller progeny cells (Figure 1D). The RNA binding protein Elav is expressed in immature neurons and differentiated primary neurons that function in the larval CNS (Figure 1E). In primary cell culture, Elav marks smaller neuronal progeny cells (Figure 1F). Antibodies for cell adhesion molecules can be used to reveal the morphology of axon tracts and dendritic extensions of differentiating neurons. The adhesion molecule Fasciclin II (FasII) marks cell bodies and pioneering axons as well as neurites in the mushroom body [18] (Figure 1G). In cell culture anti-FasII stainings outline cell bodies and axon tracts of differentiated neurons (Figure 1H).

Finally, the homeodomain transcription factor Reversed polarity (Repo) is exclusively expressed in glial cells in the larval brain [19] (Figure 1I). We found cells in primary culture that express Repo suggesting a glial cell fate (Figure 1K). In summary, commonly used molecular markers for whole mount experiments can also be used to determine cellular identity and morphology in our primary cell culture system.

### Identifying and tracing neuronal lineages in primary cell culture

**Figure 2:**
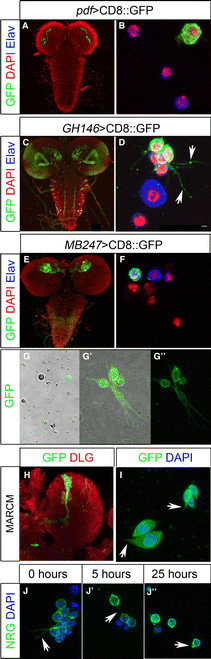
**Genetically labeled neurons in third instar larval brains and primary cell cultures.** Fixed (**A-F**, **H-J”**) and live samples (**G-G”**). Anti-Elav (blue) labels nuclei of neurons in culture (B, D, F), anti-GFP (green) labels genetically identifiable cells and DAPI shows DNA. (A, B) Expression of UAS-*CD8::GFP* driven by *pdf*-Gal4 (*pdf* >*CD8::GFP*) reveals the eight LNs in the third instar brain (A) and in primary cell culture (B). (C, D) *GH146* > *CD8::GFP* shows GFP-expression (green) in projection neurons of the antennal lobe and the developing optic ganglia (C) and in primary cell culture (D) (arrows indicate outgrowing neurites) (E, F) *MB247* > *CD8::GFP* labels Kenyon cells in the mushroom bodies of the larval brain (E) and in cultured cells (F). (G, G’, G”) DIC images overlaid with confocal images shows living GFP-expressing cultured neurons and their neurite extensions (G’ is an overview, G’ and G” are higher magnifications). (H) Shown are secondary neurons in a mCD8::GFP labeled MARCM clone. Brain tissue is stained with anti-Dlg (red) (I) Dissociated secondary neurons reveal small extensions in culture. J, J’, J’’) Cells plated on Concanavalin A coated slides show neuronal extensions. Neurites are still visible after 5 h but have largely disappeared at 25 h after plating. Cell morphology is given by anti-Nrg staining (green).

The Gal4/UAS system [20] allows us to genetically identify and manipulate particular neuronal lineages in the developing brain. Gal4 driven membrane tethered *mCD8::GFP* is used to visualize morphology and arborizations of neuronal cells to describe neuronal circuits. We tested whether the GAL4/UAS system can be applied to identify and trace particular neuronal lineages or neuron subtypes in primary cell culture. We used three specific Gal4 driver lines to induce mCD8::GFP in distinct neuronal lineages in the larval brain. *pdf*-Gal4 is specifically expressed in the eight main pace maker neurons of the clock circuit [21,22] (Figure 2A). *GH146*-Gal4 is expressed in projection neurons of the antennal lobe and the developing optic ganglia [23,24] (Figure 2C) and *MB247*-Gal4 is expressed in the Kenyon cells of the mushroom bodies [25-27] (Figure 2E). As expected in primary cell cultures derived from *pdf*-Gal4/UAS-m*CD8::GFP* animals, we found a small number of GFP positive cells (Figure 2B). Since only eight cells per brain are GFP-positive, this finding shows that even rare cell types can be studied in primary cell culture. In cultures from *GH146*-Gal4/UAS-m*CD8::GFP* and *MB247*-Gal4/UAS-m*CD8*::*GFP* animals, we found GFP expressing cells (Figure 2D, F) and neurite extensions can be observed in differentiating neurons (Figure 2D).

We also tested whether we can use the GAL4/UAS system to trace neuronal lineages in unfixed cultured neurons. We were able to visualize GFP positive neurons with axonal extensions in the living primary culture. This suggests that the brain dissociation protocol can be used in combination with the GAL4/UAS system to perform lineage analysis in fixed culture and live imaging experiments (Figure 2G, G’, G’’).

We observed neurite extensions in *GH146*-Gal4/*UAS-mCD8::GFP* and *MB247*-Gal4/*UAS-*m*CD8*::*GFP* primary cell cultures. These cells are likely primary neurons of embryonic origin. To address if secondary neurons are able to form neurite extensions, we generated labeled clones induced during larval stages. While secondary neurons in vivo can have long extensions (Figure 2H), we found that mCD8::GFP labeled cells in culture are able to form small cellular extensions (Figure 2I). We next addressed how neurite extensions behave over time. We cultured wildtype brain cells and visualized cell extensions with an antibody against the cell adhesion molecule Neuroglian (Nrg). After dissociation and plating cells on a Concanavalin A coated slide, we observed many cells with a neuronal morphology showing neurite extensions (Figure 2J). After 5 h cells still show neurite extensions; however, after 25 h, cellular projections are largely diminished (Figure 2J’, J”). Hence, initial outgrowth of extensions is established but not maintained over longer time periods.

### Identifying mitotically active cells in primary cell culture

**Figure 3:**
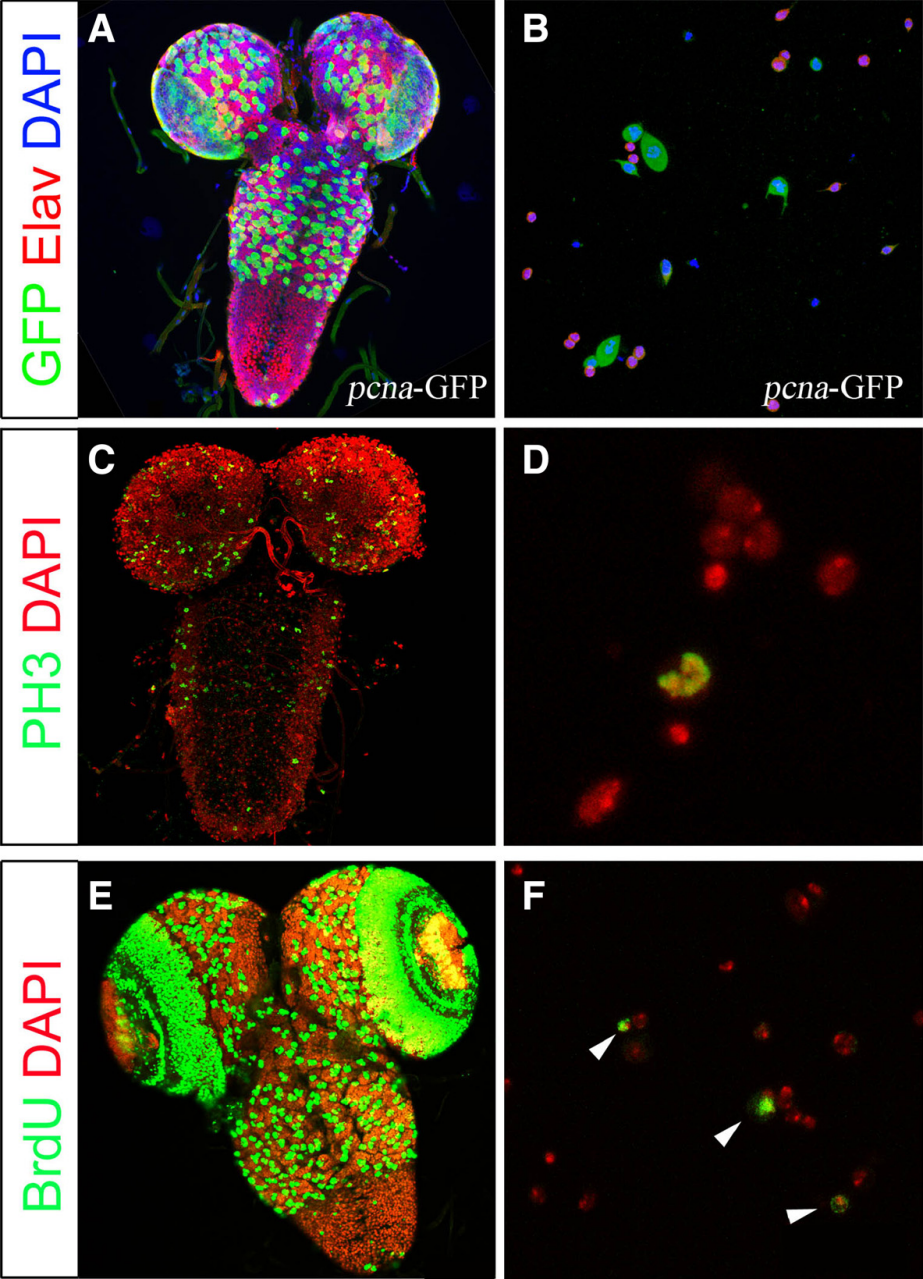
**Proliferation and cell division markers in third instar larval brains and primary cell cultures.** (**A, B**) The *pcna*-GFP line positively-labels cells in S-phase (green) co-labeled with anti-Elav (red) and DAPI. (**C, D**) Immunostaining with anti-phosphorylated Histone3 antibody (PH3, green) shows mitotic cells in larval brains (C) and in culture (D). (**E, F**) BrdU (green) pulse for 3.5 h in third instar larval brains (E) and 4-h-pulse in primary culture mark proliferating cells, that have replicated DNA (F; arrows).

Spatially and temporally controlled proliferation of neural precursors in the brain is a key process to generate the correct neuron and glial number during defined developmental time windows. Mutations that affect NB proliferation can result in tumorous overgrowth or conversely, to reduced NBs lineages [1]. We therefore tested commonly used reagents, to assess proliferation patterns and mitotic activity in primary neural cell culture. We first used a construct that expresses GFP under the control of the proliferating cell nuclear antigen (PCNA) promoter [28]. *pcna*-GFP is induced in the late S-phase of the cell cycle and thus labels cycling NBs and GMCs as well as precursor cells of the developing optic lobes (Figure 3A). *pcna*-GFP expression is detected in large cells in primary cell culture, suggesting a NB identity, as well as in smaller cells, which are most likely GMCs or smaller NBs that can be found in the optic lobes (compare Figure 1G); Elav-expressing neurons (Figure 3B) do not express *pcna*-GFP, which indicates that they are post-mitotic.

Antibodies against phosphorylated Histone3 (PH3) are widely used to identify mitotically active cells in *Drosophila* and other model organisms. PH3 immuno-positive cells are found in areas of the developing larval brain (Figure 3C) where mitosis occurs. Similarly, in primary cell culture we can use anti-PH3 antibodies to visualize mitotic active cells, such as NBs and GMCs (Figure 3D).

The thymidine homolog BrdU can be used to identify cells that go through S-phase and thus actively replicate DNA. Exposure of third instar larval brains to short pulses of BrdU, results in BrdU incorporation in dividing NBs and clusters of progeny cells in the central brain and thorax, as well as in the proliferation centers of the optic lobes (Figure 3E). BrdU incorporation was found in small groups of cells in culture (Figure 3F, arrow). We conclude that molecular labeling techniques to identify mitotically active cells in whole mount larval brains can also be applied to study neurogenesis in primary neural cell culture.

### Neural precursors reveal mitotic activity and apoptosis during several days in culture

**Figure 4:**
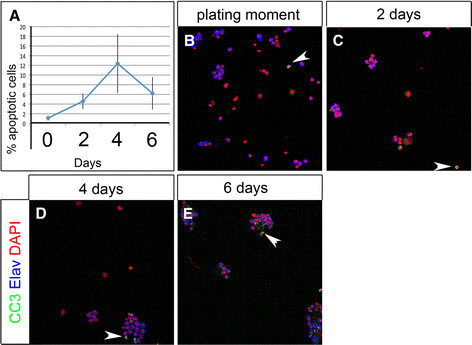
**Temporal quantification of apoptotic activity in primary cell culture. (A)** Quantification of apoptotic cells at time points displayed in B-E. **(B-E)** Immunostaining of anti-cleaved Caspase 3 (CC3, green) co-labeled with anti-Elav (blue) and DAPI (red) in (B); zero-day old, (C) two-day old, (D) four-day old, (E) six-day old cells. (B-E) Arrowheads show CC3 positive cells. (A) Percentages of apoptotic cells detected at the plating moment 1.11%, SD 0.44; Day 2: 4.56%, SD 1.61; Day 4: 12.37%, SD 6.04; Day 6: 6.22%, SD 3.34%.

**Figure 5:**
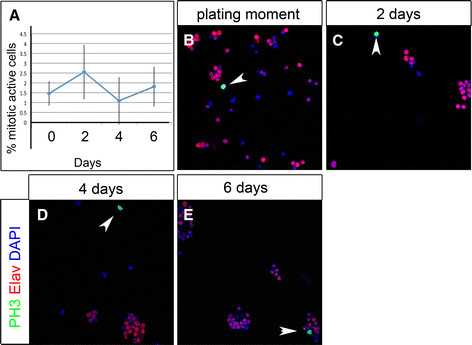
**Temporal quantification of mitotic activity in primary cell culture. (A)** Quantification of mitotically active cells at time points displayed in B-E. **(B-E)** Immunostaining of anti-phosphorylated Histone3 (PH3, green) co-labeled with anti-Elav (blue) and DAPI (red) in (B); zero-day old, (C) two-day old, (D) four-day old, (E) six-day old cells. (B-E) Arrowheads show CC3 positive cells. (A) Percentages of mitotic cells detected at the plating moment 1.46%, SD 0.62; Day 2: 2.55%, SD 1.38, Day 4: 1.09%, SD 1.2; Day 6: 1.81%, SD1.01%.

We tested in our cell culture system whether we can observe mitotic activity and apoptosis over a longer time period. We dissociated wandering third instar brains and maintained them in culture at 25°C for up to six days to analyze mitotic activity and apoptosis. Apoptotic nuclei were observed in culture from Day 0, as marked by an antibody staining for cleaved Caspase3 (CC3; Figure 4B). We assessed the number of apoptotic cells at two, four and six days after culturing (Figure 4A, C-E). At all time points we observed a percentage of apoptotic cells from 1.1% to 12.4% (Figure 4A; Day 0: 1.11%, SD 0.44; Day 2: 4.56%, SD 1.61 Day 4: 12.37%, SD 6.04; Day 6: 6.22%, SD 3.34). Over the same culture period we also analyzed mitotic activity by using an antibody against phosphorylated Histone 3 (anti-PH3) (Figure 5A-E). We found fractions of cultured cells in mitosis from 1.5% to 2.6% of total cells (Figure 5A) (Day 0: 1.46%, SD 0.62; Day 2: 2.55%, SD 1.38, Day 4: 1.09%, SD 1.2; Day 6: 1.81%, SD1.01%). Hence, we were able to monitor neural mitotic activity as well as apoptotic cell death in primary cell culture over a period of six days.

### Symmetric dividing optic lobe precursors are maintained in culture

**Figure 6:**
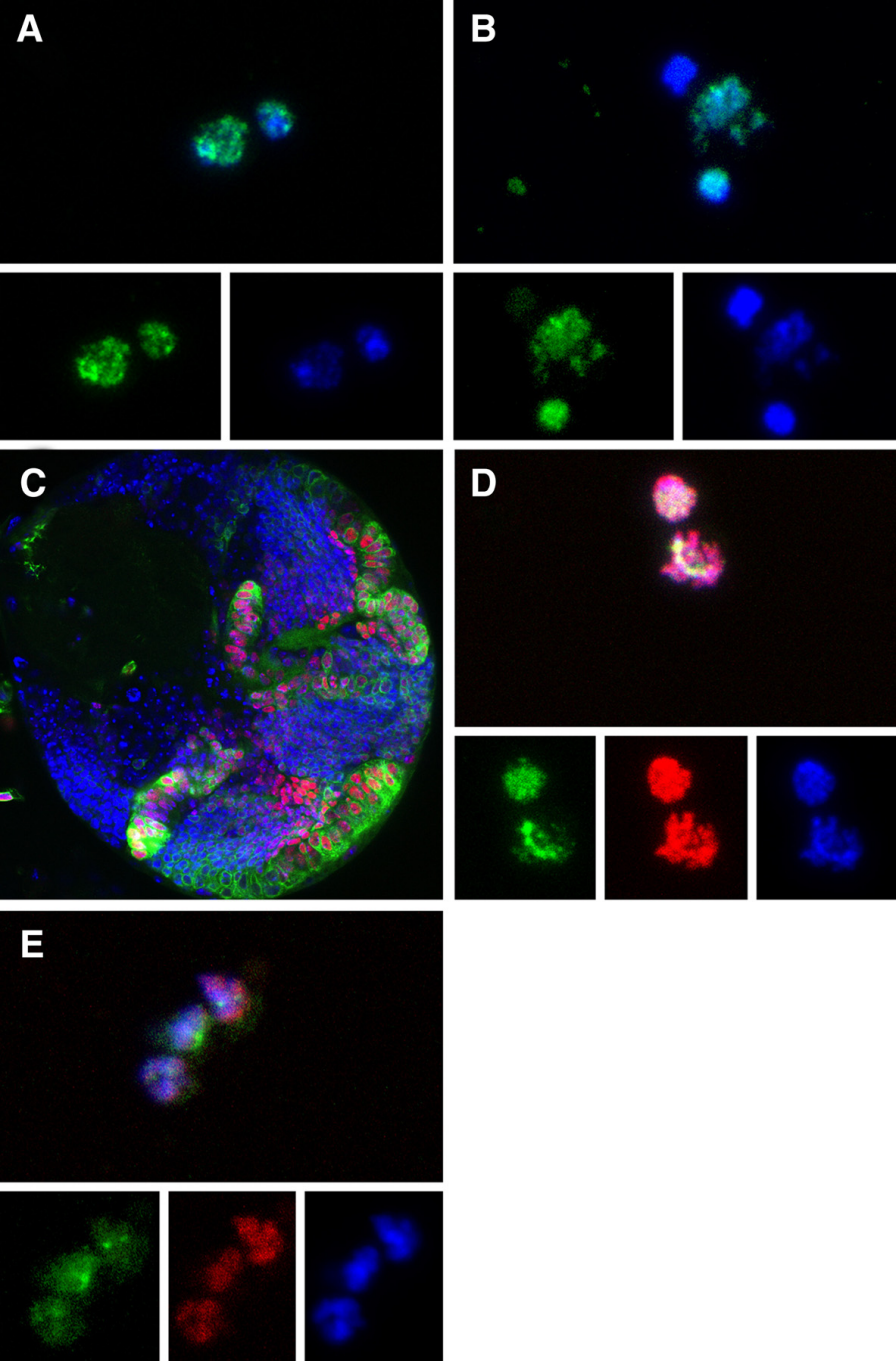
**Symmetrically dividing optic lobe precursors are maintained in culture. (A, B)** Proliferating cells shown by BrdU incorporation (green) and DAPI staining (blue), after 15 h in culture. Shown are doublets containing cells of equal size (A) and unequal cell size (B). (C) Larval brain lobe with, *c885-*Gal4 driven UAS*-mCD8::GFP,* UAS*-H2B::RFP* (red). **(D, E)** Shown are a doublet (D) and a triplet (E) of cells with incorporated BrdU (green), that are also positive for H2B::RFP (red) Dapi (blue).

We next characterized the proliferation pattern of cells in culture in greater detail. Dissociated cells in culture were incubated with BrdU and examined after 15 h and 45 h in culture. We found that after 15 h 6.9% of total cells (n = 1,201) have gone through S-phase and reveal incorporated BrdU. At 45 h in culture 8% of total cells (n = 1,051) have gone through S-phase. A large fraction of BrdU + cells are singlets (78% of BrdU + cells at 15 h; 64% of BrdU + cells at 45 h). Most other BrdU + cells are clustered in doublets, indicating one cell division (Figure 6A, B) (19% of BrdU + cells at 15 h; 28% of BrdU + cells at 45 h). We only found a small fraction of BrdU + cells in triplets or quadruplets. A great majority of cells that form doublets, triplets and quadruplets are equal in size (Figure 6A). Asymmetric NB/GMC BrdU + doublets, that consist of cells with unequal size, were also observed but much less frequent (6% of BrdU + doublets) (Figure 6B). Therefore, based on size, a majority of proliferative precursor cells may undergo symmetric divisions in our culture system.

In the larval brain, symmetric divisions are found in the optic proliferation centers, where neuroepithelial cells proliferate (Figure 6C). We therefore dissociated larval brains and applied BrdU to cell culture, where optic neuroepithelial cells were genetically labeled by Histone2B::RFP (*c855a-*Gal4 driven *UAS-*H2B::mRFP). We found that at both time points about half of all BrdU + cells were also expressing Histone2B::RFP (56% at 15 h, n = 62; 49% at 45 h n = 49) (Figure 6D, E). These results suggest that half of the number of dividing cells are of optic lobe origin and are symmetrically dividing neuroepithelial cells. The other symmetric dividing cells may be GMCs and/or intermediate progenitor cells of the Type II NB lineages. Although intermediate progenitors divisions are asymmetric, based on cell fate determinant distributions, they appear symmetrically based on cell size.

### Notch misexpression leads to increased mitotic activity that is maintained in cell culture

**Figure 7:**
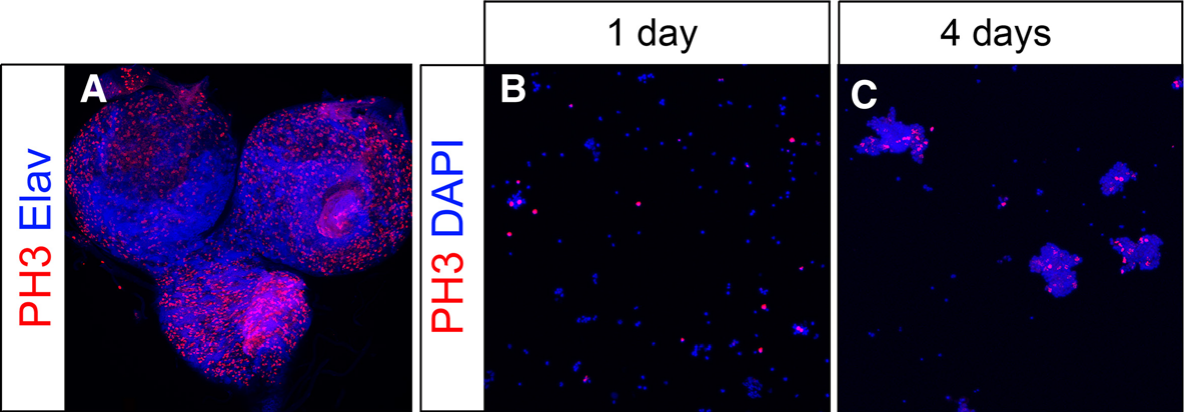
**Notch misexpression leads to increased mitotic activity in culture. (A)** Third instar larval brain of genotype *insc*-Gal4/+; *tub*-Gal80^ts^/UAS*-Notch*^*intra*^ stained with anti-PH3 (red) and anti-Elav (blue). **(B, C)** Cell culture of genotype insc-**Gal4**/+; tub-**Gal80**_**ts**_/**UAS**-Notch_intra_ stained with anti-PH3 (red) and DAPI (blue). Cells maintained for one day (B) and four days (C) in culture.

Previous studies have shown that misexpression of the intracellular domain of Notch (Notch^intra^) leads to tumorous overgrowth in larval NB lineages [5,29-32]. We therefore investigated whether overproliferating precursors, deriving from tumorous brains, can be maintained in vitro. To address this question, we misexpressed *Notch*^*intra*^ during larval brain development by using the NB specific driver line *insc*-Gal4 [33]. To control the onset of Notch misexpression, we used the temperature sensitive GAL4 inhibitor GAL80^ts^. Embryos were collected at 18°C and freshly hatched larvae were shifted to 29°C, to restrict Notch misexpression to larval stages. Primary cell cultures were prepared from wandering third instar brains that showed tumorous overgrowth (Figure 7A). In one-day-old cultures we observed an increased number of PH3 positive cells (8.3% n = 1,452), as compared to wildtype (1.5 to 2.6% see above) (Figure 7B). Thus, cultures from Notch misexpression tumorous brains reveal a three- to five-fold increase in the number of mitotic precursor cells. After four days in culture cell cluster have grown in size (Figure 7C). These clusters contain many PH3 positive cells, which indicate that they are mitotically active. These findings show that in contrast to normal neural precursors, the mitotic potential of neural precursors deriving from tumorous brains is markedly prolonged.

## Conclusions

The developing brain of *Drosophila* is an impacting model system, to understand how neuronal cells are specified and how they are instructed to build a functional neuronal network. In vivo studies in the developing brain have to deal with great cellular complexity and, in particular, it is difficult to distinguish between cell-autonomous and cell non-autonomous factors. To complement in vivo studies we have established a primary cell culture system, which allows us to study normal and genetically manipulated neural lineages in vitro. We were able to examine neural mitotic activity and apoptosis for six days after larval brain dissociation. Interestingly, in the living animal, neurogenesis in the brain ceases during metamorphosis and, virtually, no mitotic activity can be observed 4 days (96 h) after puparium formation [34,35]. Primary cell cultures derived from tumorous brains maintain an increased pool of mitotically active precursor cells. This may be a first step towards establishing a first stable cell line of neural precursor cells in *Drosophila*. Our cell culture system now facilitates experiments to analyze the contribution of extrinsic and intrinsic factors that control neurodevelopmental processes, such as proliferation and the termination of mitotic activity.

## Materials and methods

### Fly stocks

Flies were kept at standard laboratory conditions and raised on corn-meal medium at 25°C, 12:12 h light:dark cycle. We used the following lines: *w*^*1118*^*,* canton S (CS), *GH146-*Gal4*, pdf-*Gal4*, MB247-*Gal4*,* UAS*-CD8::GFP, pcna-*GFP, *w*; *c855a*-Gal4 (Bloomington *Drosophila* Stock Center, Indiana, USA), UAS*-Histone2B::mRFP1* (from Y. Bellaiche). For clonal induction, we used *hs-*FLP*, tub-*Gal4*,* UAS*-mCD8::GFP/CyO, FRT82B, tub-*Gal80*/TM6B* and *w; FRT82B* (from B. Bello). For Notch misexpression experiment we used *insc*-Gal4; *tub*-Gal80^ts^[33] (Bloomington) and UAS*-Notch*^*intra*^ (from S. Bray).

### In vitro primary cell culture of larval brains

Primary cell cultures were obtained from wildtype and transgenic wandering third instar larvae. The cell culture protocol was adapted and modified after [36]. The larvae were collected in PBS and their surface-sterilization achieved by immersing the larvae twice into 70% ethanol and three times into sterile water. Larval brains were dissected on sterile Petri dishes, in culture medium which is 90% L-Glutamine-supplemented Schneider`s medium (Invitrogen, Lucerne 6, Switzerland), 10% heat-inactivated Fetal Bovine Serum (HyClone Laboratories, Logan, UT, USA), 0.1% penicillin G (50–unit/ml)/streptomycin sulfate (50 μg/ml) (Gibco). The brain lobes and ventral nerve cord were collected in a saline similar to Rinaldini solution (800 mg NaCl, 20 mg KCl, 5 mg NaH_2_PO_4_H_2_O, 100 mg NaHCO_3_, 100 mg glucose, in 100 ml distilled water) and kept on ice, until the desired number of brains was collected. Dissected brains were washed three times in Rinaldini-like solution and then incubated in 0.5 mg sterile-filtered Collagenase solution type I (Sigma-Aldrich, Buchs, Switzerland)/ml Rinaldini-like solution, for 1 h, at room temperature. To remove the collagenase solution, three washes with culture medium were performed. A total of 10 μl of culture medium/brain were added and dissociation of brains into single cells was achieved by repeatedly flushing the brains through the pipette tip. For all of the protocol`s steps, we used siliconized Eppendorfs (Fisher Scientific, Wohlen, Switzerland) and pipette tips (VWR International, Dietikon, Switzerland). The cell suspension was plated into 96-well plates (40 μl cell suspension/well) (BD Falcon, Allschwil, Switzerland) and supplemented with culture medium (160 μl/well). Dissociated cells do not adhere and are free floating in culture medium. To achieve cell adhesion for cell differentiation experiments we cultured brain cell suspension on UV-treated 8-wells diagnostic Teflon slides (Thermo Scientific, Allschwil, Switzerland), coated with 15 μg sterile-filtered Concanavalin A/ml H_2_O (Sigma-Aldrich). A total of 40 μl cell suspension was placed in one well of the slide and supplemented with 40 μl culture medium. All cultures were kept in sterile wet boxes in an incubator (OKT Germany GmbH, Potsdam, Germany) at 25°C.

### Immunofluorescent stainings and antibodies

For whole mount brain immunofluorescent labeling, larval brains were dissected in PBS (Gibco, Invitrogen). Brains were fixed for 30 min in 4% formaldehyde PBS solution (Sigma-Aldrich). Brains were washed in PBS-Triton 0.3% (Acros Organics, Basel, Switzerland) three times for 10 min. Non-specific binding sites in the tissue were blocked with 10% Normal Goat Serum (NGS) (Vector Laboratories, Servion, Switzerland) diluted in PBST for 30 min. Primary and secondary antibodies were diluted in PBST and 5% NGS. Primary antibodies were incubated overnight at 4°C and secondary antibodies at room temperature for 2 h. Brains were washed four times for 15 min. Brain samples were mounted in Vectashield mounting medium with DAPI (Vector Laboratories).

Primary brain culture immunofluorescent labeling was performed in a wet box, on 15 μg Concanavalin A/ml H_2_O-coated eight-well diagnostic Teflon slides (Thermo Scientific). A total of 40 μl cell suspension was placed in one well of the slide and allowed to settle down for 15 min. During this period cells start to adhere to the coated surface in order to perform immunostainings. The cells were fixed for 15 min in 4% formaldehyde and washed with PBST three times, every 2 min. 10% NGS treatment was applied for 30 min. Immunofluorescent labeling was performed as described for whole mount larval brains.

If not stated otherwise, antibody dilutions apply for immunofluorescent labelings of whole mount brains and cell cultures: rat and mouse anti-Elav (1:30, Developmental Studies Hybridoma Bank, DSHB, Iowa City, Iowa, USA), mouse anti-Pros (1:10, DSHB), mouse anti-Repo (1:10, DSHB), mouse anti-BrdU (1:200 for brains and 1:300 for primary brain culture staining, DSHB), mouse anti-FasII (1:30 on brains and 1:10 on cells, DSHB), sheep anti-GFP (1:1000, AbD, Serotec, Kidlington, UK), mouse anti-PH3 (1:1000, Cell Signaling Technology, Allschwil, Switzerland), rabbit anti phospho-Histone H3 (1:1000, Upstate Biotechnology, Lake Placid, NY, USA), rabbit anti Cleaved Caspase3 (1:200 for brains and 1:300 for primary brain culture staining, Cell Signaling Technology), rat anti-Deadpan (8:10, gift of C.Q. Doe), guinea pig anti-Deadpan (1:500, gift of J. Skeath), mouse anti-Discs large (1:100, DSHB), mouse anti-Neuroglian/BP104 (1:30, DSHB). Fluorescent labeled secondary antibodies raised in Donkey or Goat were used (Molecular Probes, Lucerne, Switzerland and Jackson ImmunoResearch, West Grove, PA, USA) at a dilution of 1:300 for whole brains and 1:400 for cultured cells.

### BrdU labeling, clonal induction and *Notch* misexpression

Dissected L3 larval brains were incubated in 15 μg BrdU/ml of culture medium [37]. For cell culture experiments, 15 μg of BrdU/ml culture medium was added. DNA was denatured in 2 N HCl for 30 min in whole brains and for 15 min in cultured cells. BrdU incorporation was detected by immunofluorescent labeling as described above.

To induce GFP labeled clones we used the MARCM (mosaic analysis with a repressible cell marker) system [38]. Larvae with genotype *hs-*FLP*; tubP-*GAL4*, UAS-*mCD8::GFP*/+; FRT82B, tub-*GAL80*/FRT82B* were heat shocked for 1 h at 37°C and brains were dissociated at wandering third instar.

For Notch misexpression embryos of genotype w; *insc*-Gal4/+; *tub*-Gal80^ts^/UAS*-Notch*^*intra*^ developed at 18°C and freshly hatched larvae were transferred to 29°C to induce Notch gene expression in NBs. Notch misexpression cultures were prepared from wandering third instar larvae and incubated at 25°C.

### Microscopy and image processing

A Leica TCS SP5 confocal microscope was used to collect Z stacks with optical sections at 0.5 to 1.5 μm intervals. Whole brains were imaged with a 20X objective and cultured cells were imaged with a 63X oil-immersion objective. To follow GFP tagged proteins in neurons we used DIC microscopy and the LAS AF software in our confocal. The Cell Counter plugin of ImageJ was used to analyze mitotic cells and apoptotic cells in single sections. Images were processed in ImageJ and Adobe Photoshop.

## Abbreviations

ALH = After larval hatching; APF = After puparium formation; CNS = Central nervous system; GMC = Ganglion mother cell; NB = Neuroblast.

## Misc

These authors contributed equally to this work

## Competing interests

The authors declare that they have no competing interests.

## Author’s contribution

MMM, DBB and BE carried out experiments. SGS, BE and MMM designed experiments and wrote the manuscript. All authors read and approved the final manuscript.
